# Supplementary material for: The Female-Specific W Chromosomes of Birds Have Conserved Gene Contents but Are Not Feminized
Source: Genes (Basel). 2020 Sep 25;11(10):1126. doi: 10.3390/genes11101126 (PMC7599627; doi:10.3390/genes11101126)
Supplement: Supplementary file 1 [file genes-11-01126-s001.zip › supporting_materials/genes-859801-Summplementary Figures.docx]

Summplementary Figures for

The Female-Specific W Chromosomes of Birds Have Conserved Gene Contents but are Not Feminized

Luohao Xu ^1,2^ and Qi Zhou ^1,2,3,^*

^1.^ MOE Laboratory of Biosystems Homeostasis & Protection, Life Sciences Institute, Zhejiang University, Hangzhou 310058, China; luohao.xu@univie.ac.at

^2.^ Department of Neuroscience and Developmental Biology, University of Vienna, Vienna 1090, Austria

^3.^ Center for Reproductive Medicine, The 2nd Affiliated Hospital, School of Medicine, Hangzhou 310052, Zhejiang University, China

***** Correspondence: zhouqi1982@zju.edu.cn

**Figure S1.** Sequencing coverage of female reads along the Z chromosome. Female coverage was estimated using samtools depth for species with (**a)** large- or intermedian-size PARs (pseudoautosomal region) and (**b)** small PARs. Each dot represents a 50 kb window that contains at least 30 kb sites covered by reads. The X axis show the positions on the Z chromosomes. The dotted line demarcates the boundary of PAR and DR.


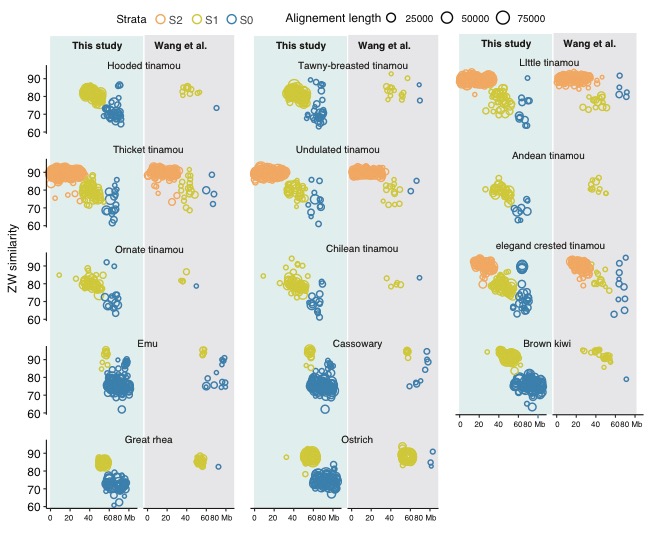


**Figure S2.** Demarcation of evolutionary strata of the paleognath sex chromosomes. We compared the Z/W sequence similarity results between this study and Wang *et al*. This study retrieved more W-linked sequences. Each dot represents a 100 kb window which was required to contain alignments with a total length larger than 4 kb. The chromosome positions (X axis) were based on the reconstructed pseudo-Z chromosome. The older strata (S0 is the oldest) shows lower Z/W sequence similarity.

**Figure S3.** The previous identified new stratum in Chilean tinamou is an artefact. The SNP density is defined as the numbers of SNP over 50 kb windows. While the previous identified new stratum exhibits a high SNP density, it’s not significantly different from that of autosomes (**b**); and when the same analysis was repeated with the re-sequencing data of another individual (Xu *et al.* 2019), the SNP density is of the ‘new stratum’ is not different from the other part of the PAR (**a**). Together, our new analysis suggested the ‘new stratum’ was an artefact and should be a PAR sequence.

**Figure S4.** The retained genes on bird W chromosomes have high ancestral expression and dosage-sensitivity. **a)** The violin plot shows the expression levels of the homologs of bird sex-linked genes in green anole lizard. The TPM value of 40 was used as a cut-off to define the high and low expression genes. The Cons and nonCons genes were defined in Figure 3c,d. (**b)** The violin plots shows the haploinsufficiency scores (Hs) of the homologs of bird sex-linked genes. The high and low dosage sensitivity were defined by the Hs of 0.4. (**c,d)** The expression levels of all sex-linked retained genes (nonCons and Cons) and the genes that were sex-linked but lost in bird W chromosome, in green anole lizard. The results from S0 and S1 are shown separately. The retained genes tend to have more highly expressed genes and dosage sensitive genes.

**Figure S5.** The retained genes on bird W chromosomes do not have more ovary dominant genes. The ovary dominant genes were defined in Materials and Methods. The Y-axis represent the proportions of ovary dominant and non-ovary dominant gene for each gene category. The dashed line represents the proportion of non-ovary dominant in autosomes. Both lost and retained (either conserved or non-conserved) genes show a similar proportion of ovary dominant genes compared with autosomes.
